# Effect of strict oral hygiene on self-rated halitosis and quality of life in patients with periodontitis: a secondary randomized controlled trial analysis

**DOI:** 10.1590/1807-3107bor-2026.vol40.021

**Published:** 2026-05-18

**Authors:** Bruno Ferraz Barbosa da Costa, Vanessa Feitosa Alves, Luísa Schertel Cassiano, Gustavo Giacomelli Nascimento, Valeska Maria Souto Paiva, Lívia Valéria Lins e Silva, Sabrina Garcia de Aquino

**Affiliations:** (a) Universidade Federal da Paraíba – UFPB, Department of Clinical and Social Dentistry, João Pessoa, PB, Brazil.; (b) Aarhus University, Department of Dentistry and Oral Health, Section for Periodontology, Aarhus, Denmark.; (c) National Dental Research Institute Singapore, National Dental Centre Singapore, Singapore, Singapore.

**Keywords:** Halitosis, Oral Hygiene, Periodontitis, Quality of Life

## Abstract

This single-blind, randomized clinical trial evaluated the impact of a strict oral hygiene (OH) phase on self-reported halitosis and quality of life (QoL) in patients with periodontitis. This study reports a secondary analysis of a single-blind randomized controlled trial (parallel, 1:1), registered at RBR-5jmx32v. The study included 24 individuals diagnosed with periodontitis who were randomly assigned to the intervention group (IG; n = 12) and the no-intervention group (NIG; n = 12). After receiving personalized OH instructions, the IG participants followed a rigorous OH phase for 45 days, whereas the NIG participants received no guidance during this period. The halitosis-associated QoL test (HALT) was administered at baseline and after 45 days. Data were analyzed using t-tests, chi-square tests, and multilevel linear regression, with significance set at p < 0.05. The average age of the study participants was 47.2 years, with a balanced sex distribution and no differences in income or education. Self-reported halitosis decreased from 100% to 50% in the IG after 45 days, whereas it remained at 91.6% in the NIG (p = 0.034). The IG also showed a mean HALT score reduction of 11.9 points (−11.88; 95% CI: −19.40 to −4.36), with significant improvements in social impact and self-esteem (p < 0.05). Clinically, BOP (p < 0.001) and VPI (p = 0.03) improved significantly in the IG, with no significant changes in NIG. This exploratory trial demonstrates that implementing a strict OH phase in patients with periodontitis may help reduce self-reported halitosis, improve HALT scores, and improve clinical outcomes, supporting its role as an adjunctive strategy in periodontal care.

## Introduction

Halitosis, an unpleasant odor from the oral cavity, affects approximately 30% of the global population and is frequently associated with periodontitis.^1^ This chronic inflammatory disease can cause attachment loss, gingival inflammation, and periodontal pockets that favor biofilm accumulation and volatile sulfur compound (VSC)-producing bacteria,^2^ the major contributor to halitosis. Halitosis negatively impacts quality of life (QoL), causing embarrassment, social isolation, and emotional distress.^3^

Proper oral hygiene (OH) relies on mechanical biofilm control through brushing, flossing, and tongue cleaning and is essential for the management of periodontitis and halitosis. The dorsum of the tongue is particularly relevant because it serves as a primary reservoir of bacteria responsible for malodor.^4,5^

The strict OH phase, which is an intensive component of periodontal therapy, stabilizes periodontal health and improves clinical parameters.^4^ This case may additionally reduce halitosis although its direct effect on QoL in patients with periodontitis still requires further investigation.

Given that halitosis also carries psychological consequences, such as anxiety and depression,^6^ patient-reported outcome measures (PROMs), including the halitosis-associated QoL test (HALT) questionnaire, are valuable tools for assessing treatment outcomes.^7^ Despite evidence supporting the benefits of the OH phase, maintaining consistent adherence to daily practices remains a significant challenge in clinical settings.^6,8^

Hence, this study aimed to evaluate the effect of a strict OH regimen on self-reported halitosis and QoL in patients with periodontitis. We hypothesize that improved adherence to OH practices will result in more favorable outcomes.

## Methods

### Ethical aspects

This study was conducted in accordance with the 1975 Declaration of Helsinki, revised in 2013,^9^ in compliance with Resolution No. 466/2012 of the National Health Council (CNS). In addition, this study was approved by the Research Ethics Committee (Report No. 4,878,043) and registered on the Brazilian Clinical Trials Registry platform under number RBR-5jmx32v. This study was conducted at the undergraduate periodontics clinic of the Federal University of Paraíba from April 2022 to February 2024.

### Study design

This study reports a secondary analysis of a single-blind, randomized controlled clinical trial. Moreover, this study was designed according to the CONSORT 2025 Statement criteria, reporting adherence to the CONSORT extension for nonpharmacologic treatments. Elements relevant to eHealth delivery were aligned with CONSORT-EHEALTH. The original trial's primary outcome was to evaluate the effect of the strict OH phase on periodontal clinical parameters in patients with periodontitis. Individuals diagnosed with periodontitis were randomly assigned to two groups: no intervention (NIG) and intervention (IG) groups. Participants in the IG received individualized motivation and guidance on OH to improve home biofilm control, whereas those in the NIG received no intervention. Both groups were re-evaluated after 45 days,^10^ during which self-reported halitosis and QoL questionnaires were administered, along with baseline assessments.

Participants underwent clinical examinations as part of the screening process at baseline, without prior knowledge of the 45-day follow-up. This approach was designed to minimize the "Hawthorne effect." In addition, the number of consultations in both groups was standardized (n = 2), ensuring that all participants experienced the same level of observation by the researcher.

### Sample size calculation

**Figure 1:**
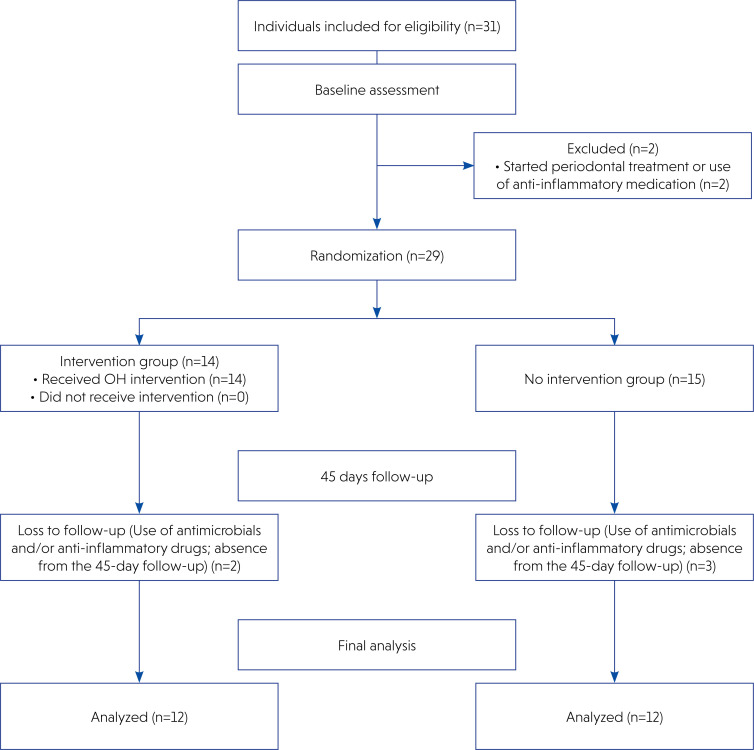
CONSORT Flow Diagram-Diagram illustrating the study's sampling process, from initial recruitment to the final data collection at 45 days. Source: Author's own work.

The sample size calculation was based on data from a previous study that evaluated the effect of the OH phase on periodontitis (Preus et al., 2020). This calculation was performed assuming a statistical power of 80%, a significance level of 5%, and an expected mean difference of 28 pockets ≥ 7 mm between the test and control groups. A minimum of 12 participants per group was estimated based on these parameters. The target sample size was adjusted to 15 participants per group to accommodate an anticipated dropout rate of 20%. Figure 1 provides a detailed overview of the recruitment process and sample progression. Accordingly, the study was completed with the minimum required sample size as initially calculated, after accounting for dropout during the study period. This study is part of a larger project. The sample size calculation was performed with the primary outcome being the reduction of periodontal pockets after the strict OH phase.

### Sample eligibility

The eligibility criteria were as follows: individuals diagnosed with periodontitis, of both genders, aged between 25 and 65 years, and with a minimum of 10 teeth in the oral cavity. In addition, only patients who reported having halitosis were included in the study. The diagnosis of periodontal condition was diagnosed by a calibrated researcher using the clinical parameters recorded on the periodontal chart.^11^ All study participants signed the Informed Consent Form. Individuals were excluded if they had a history of HIV or hepatitis, were pregnant or breastfeeding, or had used antimicrobial or anti-inflammatory medications in the 3 months before the examination. Participants were excluded if they had initiated or were undergoing periodontal or orthodontic treatment, had received prophylaxis within 6 months prior to the study, or regularly used mouthwash.

### Randomization

Randomization was stratified considering smoking habits as a stratification variable to ensure proper balance regarding relevant baseline risk factors. Allocation sequences were generated electronically using the Sealed Envelope software. A block randomization with a block size of 3 and an allocation ratio of 1:1 was applied. A team member who was not involved in data collection or outcome assessment performed allocation, and the randomization list was kept confidential. All other investigators, including those responsible for data collection and outcome evaluation, were blinded to group assignments throughout the study.

### Intervention—OH phase

**Figure 2:**
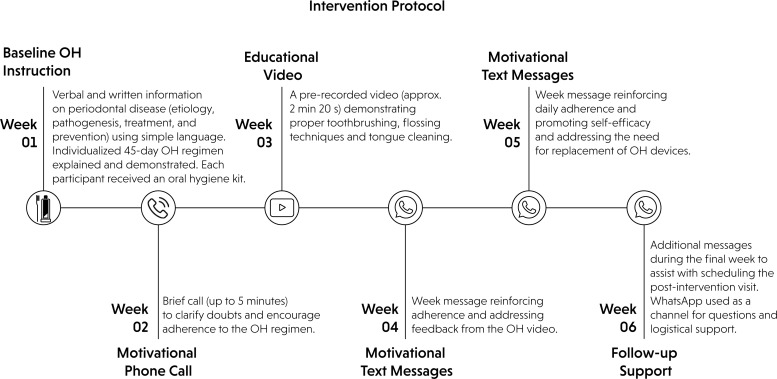
Schematic representation of the structured oral hygiene intervention delivered over a 6-week period, with passive monitoring of message delivery and reading, and participant feedback considered as qualitative indicators.

OH guidance and motivation were conducted in several stages (Figure 2) during the consultation, following a procedure similar to that described by Preus et al. (2020).^4^ In the first stage, participants were provided with verbal and written information about periodontal disease (periodontitis), including its etiology, pathogenesis, treatment, and prevention. Language appropriate to their level of understanding was used to facilitate comprehension and engagement. Next, an individualized OH regimen was explained to be followed for 45 days, considering each patient's oral condition. This information was given verbally and in writing, and each participant received an OH kit, which included the following items: an illustrated leaflet with OH instructions, a soft-bristled toothbrush with a small head, fluoridated toothpaste, dental floss or a floss pick (depending on the participant's manual dexterity), an interdental brush, a tongue scraper, and a single-tuft toothbrush when indicated.

The OH technique is demonstrated in the following stage. In addition, the patient performed it under supervision to address any questions or difficulties. The modified Bass technique is recommended for cleaning the buccal and lingual surfaces. Participants were instructed on the proper use of dental floss for interproximal surfaces, and when anatomical space allowed, the use of an interdental brush was also recommended. For tongue hygiene, the participants were instructed to use a tongue scraper with unidirectional movements from the back of the tongue toward the tip, aiming at the mechanical removal of tongue coating.

Motivational reinforcement was delivered weekly over the 45-day intervention via WhatsApp Messenger, supplemented by phone calls when needed. The protocol, although not previously validated, was based on behavior change strategies through oral health education, personalization, follow-up, and positive feedback. Weekly content included a brief motivational call (≤ 5 min), a short educational video (~2 min and 20 sec), illustrated materials with step-by-step hygiene instructions, and motivational text messages to promote adherence and self-efficacy. In the final week, reminders were sent to schedule follow-up visits. Content was standardized but allowed for personalized, bidirectional communication; participant questions were addressed individually. WhatsApp also provided logistical support (e.g., replacing materials). Engagement was passively monitored via delivery/read receipts and participant feedback. Spontaneous interactions were considered to be indicators of engagement although no tracking tools were used. This approach followed the CONSORT-EHEALTH guidelines for digital health interventions. Antibacterial mouthwashes and anti-inflammatory toothpaste were not prescribed to avoid interfering with biofilm mechanical control.

A periodontal researcher, who did not participate in data collection, conducted the guidance and motivation sessions, ensuring blinding of the study.

### No-intervention group

The participants in the no-intervention group underwent an initial clinical examination, during which questionnaires were administered and data were collected. After 45 days, the participants were invited for a new periodontal evaluation and a re-administration of the questionnaires. At this point, periodontal treatment, including OH instruction and NIG motivation, was initiated. Only participants who agreed to and signed the informed consent form were included in the study. After the 45-day study period, all NIG participants received personalized OH kits.

### Halitosis-associated life-quality test

HALT is a validated QoL questionnaire with 20 items. In this study, each item was rated on a Likert scale from 0 to 5, where 0 corresponds to "no problem" and 5 to "very severe problem." The final score is calculated by summing the scores of the 20 items, ranging from 0 to 100. A higher score indicates a greater impact on the individual's QoL. HALT includes questions related to physical, emotional, and functional limitations, as well as personal and social impairments.^12^ The questionnaire was administered to all participants in both groups at baseline and at the 45-day follow-up.

### Statistical analysis results

Multilevel mixed-effects linear regression models (mixed procedure in Stata 14.2; StataCorp, College Station, USA) were employed to estimate the association between the outcome variables—self-perception of halitosis and HALT items—in relation to the interaction between time and two groups. Sex, age, diabetes, and smoking were accounted for as covariates. This study employed a per-protocol approach for data analysis, and the researchers were responsible for data collection. Independent t-tests, chi-square/Fisher's exact tests, and repeated measures ANOVA were also conducted.

## RESULTS

### Sample characterization

The participants’ mean age was 47.2 years, with a balanced distribution of sex, education, and income, and no statistically significant differences between groups (p > 0.05). The distribution of periodontitis stages is also shown in Table 1.

**Table 1:** Baseline sociodemographic and periodontal characteristics of participants by study group.

HALT Items	Area of influence on quality of life
Q1. Mouth breathing	Physiological changes
Q2. Throat infection/tonsillitis	Physiological changes
Q3. Sinus infection/sinusitis	Physiological changes
Q4. Insecurity about breath	Emotional and self-esteem limitations
Q5. Tension regarding bad breath	Emotional and self-esteem limitations
Q6. Difficulty chewing or limited intake of certain foods due to bad breath	Physiological changes
Q7. Taste alteration	Physiological changes
Q8. Speech problems (or covering the mouth) due to bad breath	Physiological changes
Q9. Appearance affected due to bad breath	Emotional and self-esteem limitations
Q10. Depression due to bad breath	Social disability
Q11. Difficulty concentrating due to bad breath	Physiological changes
Q12. Shame due to bad breath	Emotional and self-esteem limitations
Q13. Time consumption due to bad breath	Emotional and self-esteem limitations
Q14. Keeping distance during conversations due to bad breath	Emotional and self-esteem limitations
Q15. Avoiding going out due to bad breath	Social disability
Q16. Communication problems due to bad breath	Social disability
Q17. People mention/imply that I have bad breath	Social disability
Q18. Financial loss due to bad breath	Social disability
Q19. Social suffering/personal loss due to bad breath	Social disability
Q20. Dissatisfaction with life due to halitosis	Emotional and self-esteem limitations

Continuous data are expressed as mean (standard deviation). The p-values reflect comparisons between the intervention and no intervention groups (£ Independent t-test; ¥ Chi-square or Fisher's exact test).

### Hygiene habits

Table 2 shows no statistical difference in OH habits between the intervention group (IG) and the no-intervention group (NIG) at baseline and at the 45-day follow-up. The frequency of daily brushing, interdental cleaning, and dental floss use was considered. No statistically significant change was observed (p > 0.05). However, participants in both groups improved their OH behavior, particularly the frequency of interdental cleaning ≥ 2 times/day, which increased in the IG from 25.0% (n = 3) to 58.3% (n = 7).

**Table 2:** Oral hygiene behavior at baseline and after 45 days of follow-up.

Oral hygiene behavior	Baseline		p-value	45 days		p-value
	Intervention	No intervention		Intervention	No intervention	
	% (n)	% (n)		% (n)	% (n)	
Brushing frequency 1x/day	8.3 (1)	16.7 (2)	0.537	0 (0)	16.7 (2)	0.336
Brushing frequency ≥ 2x/day	91.6 (11)	83.3 (10)		100 (12)	83.3 (10)	
Interdental cleaning frequency - Never	8.3 (1)	8.3 (1)	0.315	0 (0)	0 (0)	0.335
Interdental cleaning frequency - Up to 1x/day	66.7 (8)	58.3 (7)		41.7 (5)	50.0 (6)	
Interdental cleaning frequency ≥ 2x/day	25.0 (3)	33.3 (4)		58.3 (7)	50.0 (6)	
Flossing frequency (mean (SD))	4.17 (2.65)	4.83 (2.86)	0.560	5.58 (1.37)	5.50 (1.98)	0.906

The number of volunteers per group was 12. Fisher's exact test was used when the Chi-square test assumptions were violated, such as when cell frequencies were lower than 5. The p-values presented for the "flossing frequency" variable represent the simple effects of repeated measures ANOVA.

### Clinical indicators

**Figure 3:**
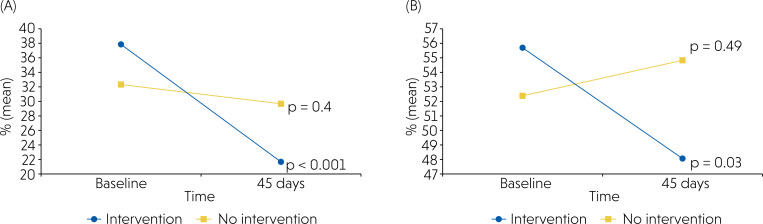
(A) Bleeding on Probing (BOP %) at baseline and after 45 days in the intervention and no-intervention groups. The intervention group showed a significant reduction in BOP from baseline to 45 days (p < 0.001), while no significant change was observed in the no-intervention group (p = 0.40). Between-group differences at 45 days were not statistically significant (p = 0.10) (B) Visible Plaque Index (VPI %) at baseline and after 45 days in the intervention and no-intervention groups. The intervention group demonstrated a significant reduction in VPI over the 45-day period (p = 0.03), whereas the no-intervention group remained stable (p = 0.49). Between-group differences at 45 days were not statistically significant (p = 0.39).

No statistically significant differences were observed at baseline between the BOP or VPI groups (p > 0.05). After 45 days, the IG showed a marked reduction in BOP (from 37.8% to 21.6%), whereas the NIG remained nearly unchanged (32.3% to 29.7%), with a borderline between-group difference (p = 0.10). Regarding VPI, the IG exhibited a modest reduction (from 55.7% to 48.0%), whereas the NIG slightly increased (52.4% to 54.8%), although without statistical significance (p = 0.39). These results are illustrated in Figure 3 (A: BOP; B: VPI).

### Self-reported halitosis

Self-perceived halitosis was assessed at baseline and after 45 days using a question with responses ranging from 0 (no halitosis) to 3 (frequent halitosis). All of the participants in both groups reported having bad breath at baseline. After 45 days, this percentage decreased to 50.0% in the IG but remained high at 91.6% in the NIG (p = 0.034; Table 3). Multilevel regression confirmed a significant reduction in self-reported halitosis in the IG (coefficient: −1.19, 95%CI: −1.84 to −0.52, p < 0.001), whereas the NIG showed no significant change (−0.33, 95%CI: −0.76 to 0.09, p = 0.129; Table 4).

**Table 3:** Self-perception of halitosis at baseline and after 45 days of follow-up.

Self-perception of halitosis	Baseline		p-value	45 days		p-value
	Intervention	No intervention		Intervention	No intervention	
	% (n)	% (n)		% (n)	% (n)	
Do you think you have bad breath?	Yes: 100 (12)	Yes: 100 (12)		50 (6)	91.6 (11)	0.034
	0	0		50 (6)	8.3 (1)	

The number of volunteers per group was 12. Fisher's exact test was used when the Chi-square test assumptions were violated, such as when cell frequencies were lower than 5.

**Table 4:** Multilevel mixed-effects linear regression of self-reported halitosis, modeled by the mean difference between baseline and follow-up.

Self-perception of halitosis	Coefficient	95%CI	p-value
Intervention	−1.19	−1.84, −0.52	0.000
No intervention	−0.33	−0.76, 0.09	0.129

The values presented correspond to the coefficients of the multilevel mixed-effects linear regression, accompanied by 95% confidence intervals (95%CI) and p-values. The table only shows the items that presented a statistically significant difference for the intervention group (IG).

### Halt

The multilevel linear regression analysis (Table 5) of HALT data showed a statistically significant improvement (p < 0.05) in the total IG score after 45 days (−11.88 [−19.40, −4.36]). Individual question analysis (Table 6) revealed significant differences (p < 0.05) for questions 1, 2, 4, 5, 6, 10, and 17. These questions fall into three categories,^14^ affecting QoL: physiological changes (1, 2, 6), emotional and self-esteem limitations (4, 5), and social disability (10, 17). The intervention showed a positive effect on reducing scores across all three categories without a predominant impact on any specific area (Table 7).

**Table 5:** Multilevel mixed-effects linear regression of statistically significant halitosis-related items, modeled by the mean difference between baseline and follow-up.

Item	Group	Coefficient	95%CI	p-value
HALT total score	Intervention	−11.88	−19.40, −4.36	0.002
	No intervention	−5.31	−11.13, 1.07	0.106
Q1. Mouth breathing	Intervention	−1.17	−1.95, −0.38	0.003
	No intervention	−0.69	−1.41, 0.03	0.061
Q2. Throat infection/tonsillitis	Intervention	−0.35	−0.69, −0.01	0.044
	No intervention	−0.16	−0.39, 0.07	0.017
Q4. Insecurity about breath	Intervention	−1.02	−1.80, −0.23	0.011
	No intervention	−0.34	−0.78, 0.09	0.123
Q5. Tension regarding bad breath	Intervention	−1.00	−1.77, −0.23	0.010
	No intervention	−0.30	−0.84, 0.23	0.275
Q6. Difficulty chewing or limited intake of certain foods due to bad breath	Intervention	−0.71	−1.29, −0.14	0.014
	No intervention	−0.09	−0.83, 0.65	0.814
Q10. Depression due to bad breath	Intervention	−0.76	−1.44, −0.07	0.030
	No intervention	−0.01	−0.04, 0.01	0.286
Q17. People mention/imply that I have bad breath	Intervention	−0.59	−1.10, −0.07	0.025
	No intervention	−0.30	−0.82, 0.22	0.258

The values presented correspond to the coefficients of the multilevel mixed-effects linear regression, accompanied by 95% confidence intervals (95%CI) and p-values. The table only shows the items that presented a statistically significant difference for the intervention group (IG).

**Table 6:** Multilevel mixed-effects linear regression of halitosis-related items, modeled by the mean difference between baseline and follow-up.

Variables		Coefficient	95%CI	p-value		Coefficient	95%CI	p-value
Halitosis						Q10			
	Intervention	−1.19	−1.84, −0.52	0.000	Intervention	−0.76	−1.44, −0.07	0.030
	No intervention	−0.33	−0.76, 0.09	0.129	No intervention	−0.01	−0.04, 0.01	0.286
HALI						Q11			
	Intervention	−11.88	−19.40, −4.36	0.002	Intervention	−0.37	−1.03, 0.28	0.263
	No intervention	−5.31	−11.13, 1.07	0.106	No intervention	0.09	−0.07, 0.26	0.261
Q1						Q12			
	Intervention	−1.17	−1.95, −0.38	0.003	Intervention	−0.47	−1.39, 0.44	0.313
	No intervention	−0.69	−1.41, 0.03	0.061	No intervention	−0.26	−0.71, 0.18	0.241
Q2						Q13			
	Intervention	−0.35	−0.69, −0.01	0.044	Intervention	0.35	−0.55, 1.25	0.449
	No intervention	−0.16	−0.39, 0.07	0.017	No intervention	0.04	−0.52, 0.62	0.871
Q3						Q14			
	Intervention	−0.76	−1.58, 0.05	0.068	Intervention	−0.31	−1.23, 0.60	0.501
	No intervention	0.09	−0.07, 0.27	0.275	No intervention	0.10	−0.62, 0.82	0.785
Q4						Q15			
	Intervention	−1.02	−1.80, −0.23	0.011	Intervention	−0.10	−0.48, 0.27	0.595
	No intervention	−0.34	−0.78, 0.09	0.123	No intervention	−0.07	−0.37, 0.22	0.631
Q5						Q16			
	Intervention	−1.00	−1.77, −0.23	0.010	Intervention	−0.32	−0.89, 0.25	0.277
	No intervention	−0.30	−0.84, 0.23	0.275	No intervention	−0.16	−0.40, 0.06	0.158
Q6						Q17			
	Intervention	−0.71	−1.29, −0.14	0.014	Intervention	−0.59	−1.10, −0.07	0.025
	No intervention	−0.09	−0.83, 0.65	0.814	No intervention	−0.30	−0.82, 0.22	0.258
7						Q18			
	Intervention	−0.25	−0.61, 0.10	0.164	Intervention	−0.03	−0.17, 0.10	0.615
	No intervention	0.33	−0.30, 0.97	0.308	No intervention	0.00	−0.01, 0.01	0.974
Q8						Q19			
	Intervention	−0.19	−0.97, 0.58	0.619	Intervention	0.01	−0.10, 0.13	0.23
	No intervention	−0.12	−0.78, 0.53	0.719	No intervention	0.09	−0.07, 0.26	1.13
Q9						Q20			
	Intervention	−0.50	−1.18, 0.17	0.142	Intervention	−0.46	−1.16, 0.23	0.191
	No intervention	−0.41	−0.87, 0.04	0.078	No intervention	−0.21	−1.17, 0.74	0.663

The values presented correspond to the coefficients of the multilevel mixed-effects linear regression, accompanied by 95% confidence intervals (95%CI) and p-values. Results with p < 0.05 were considered statistically significant

**Table 7:** HALT questionnaire divided by areas of influence on quality of life.

HALT Items	Area of influence on quality of life
Q1. Mouth breathing	Physiological changes
Q2. Throat infection/tonsillitis	Physiological changes
Q3. Sinus infection/sinusitis	Physiological changes
Q4. Insecurity about breath	Emotional and self-esteem limitations
Q5. Tension regarding bad breath	Emotional and self-esteem limitations
Q6. Difficulty chewing or limited intake of certain foods due to bad breath	Physiological changes
Q7. Taste alteration	Physiological changes
Q8. Speech problems (or covering the mouth) due to bad breath	Physiological changes
Q9. Appearance affected due to bad breath	Emotional and self-esteem limitations
Q10. Depression due to bad breath	Social disability
Q11. Difficulty concentrating due to bad breath	Physiological changes
Q12. Shame due to bad breath	Emotional and self-esteem limitations
Q13. Time consumption due to bad breath	Emotional and self-esteem limitations
Q14. Keeping distance during conversations due to bad breath	Emotional and self-esteem limitations
Q15. Avoiding going out due to bad breath	Social disability
Q16. Communication problems due to bad breath	Social disability
Q17. People mention/imply that I have bad breath	Social disability
Q18. Financial loss due to bad breath	Social disability
Q19. Social suffering/personal loss due to bad breath	Social disability
Q20. Dissatisfaction with life due to halitosis	Emotional and self-esteem limitations

The HALT questionnaire items categorized by their impact on different aspects of quality of life. The areas of influence include physiological changes, emotional and self-esteem limitations, and social disability. Adapted from Kizhner, Xu, Krespi.^12^

## Discussion

The evaluation of OHRQoL and PROMs,^7^ including clinical data, is crucial in clinical trials.^13^ This study assessed the effect of a strict OH phase on self-reported halitosis and QoL in individuals with periodontitis.

The implementation of a strict OH phase resulted in significant benefits.^4^ In the IG, self-reported halitosis decreased, with improved HALT scores, particularly in social and psychological domains, accompanied by reductions in BOP and VPI. By contrast, the NIG showed minimal change.^4^ Both groups increased OH habits, likely influenced by trial participation.^14^ However, only the IG achieved clinically relevant changes, underscoring the importance of structured and supervised OH as a primary strategy in the management of periodontitis and halitosis.

Objective measures, such as sulfur monitors and gas chromatography, provide precision. However, this study focused on the psychosocial impact of self-perceived halitosis using the HALT questionnaire,^8,15^ thereby determining emotional, social, and functional consequences relevant to the individuals themselves.^8^ This subjective approach, particularly in a condition such as halitosis, is preferred over a normative assessment based on the dentist's perception only. Organoleptic assessment and VSC measurements are considered more reliable. However, studies showed no significant differences compared with self-reporting.^16^ Self-reported conditions often influence OHRQoL more than clinical findings,^8,15^ shaping behaviors, including seeking professional care.

Most participants were diagnosed at stages III–IV of periodontitis.^11^ Patients with severe periodontitis experience worse QoL because of functional and social impacts.^17^ Indeed, chronic inflammation with tissue destruction promotes biofilm accumulation, increasing halitosis risk.^18^ Consider the slow progression of periodontitis in approximately 90% of adults, as shown by the natural history of periodontitis studies (average attachment loss 0.3 mm/yr).^19^ In this case, our decision to postpone the strict OH phase in the NIG group is unlikely to cause any harm to this group, particularly considering that similar instructions were given after the trial period.

Mechanical plaque control is central to treatment and relies on personalized motivation and guidance owing to the impact of biofilm on periodontitis and halitosis.^4^ Although some studies recommend multiple sessions,^20^ our study used a single in-office instruction, following the recent approaches,^4^ to minimize the Hawthorne effect and foster patient engagement.^21^ Considering that changes in OH habits may take weeks and cause temporary discomfort, weekly messages and videos were provided to sustain motivation in IG.^22,23^ The intervention was designed as an integrated set of strategies, with adherence and clinical benefits attributed to their combined effect.^23^

Although hygiene habits did not significantly differ between groups, both increased interdental cleaning. The NIG may have improved OH habits because of the Hawthorne effect, despite not receiving formal guidance.^14^ Participation in health research alone may increase awareness and promote healthier behaviors, possibly explaining the similar improvements of the NIG to the IG.^24^ The sample's socioeconomic and geographic homogeneity led to similar baseline behaviors, whereas the limited sample size reduced the statistical power to detect significant post-intervention changes.

Nevertheless, our study demonstrated that patient-driven biofilm control, even without dentist intervention, can improve self-reported halitosis. This approach improved OHRQoL positively and can serve as a valuable motivational tool in periodontal treatment and oral self-care.^8,26^ These benefits may not only facilitate adherence to subsequent treatment but also prevent complications during periodontal treatment and promote a continuous cycle of self-care.^27^ These improvements likely reflect reduced inflammation^4^ and changes in microbiota after the strict OH phase, as evidenced by significant BOP (p < 0.001) and VPI (p = 0.03) reductions in the IG. By contrast, the NIG remained stable. However, improvements in self-reported halitosis may have been primarily attributable to tongue coating removal, as highlighted in other studies.^6,28^

Self-reported information is a valid assessment tool for conditions such as halitosis, as evidenced in the literature.^8,16,29^ However, patients with halitosis often fail to recognize their own bad breath, which may be explained by olfactory desensitization. Studies have noted lower odor threshold scores in patients with halitosis.^30^ Hence, self-reported halitosis in this study may be underestimated as individuals may have difficulty detecting their own bad breath or may feel embarrassed to disclose it.^31^

The IG data showed an 11-point reduction in the mean HALT score (−11.88 [−19.40, −4.36]). The findings support the hypothesis that the strict OH phase positively impacts halitosis in patients with periodontitis.^32,33^ Given the relationship between HALT and QoL, the personalized OH protocol may improve the QoL of IG patients by reducing insecurity and anxiety about bad breath.^34^ Interestingly, a recent clinical study using laser tongue debridement as an adjunctive therapy for halitosis reported a nine-point reduction in HALT scores after the intervention, further supporting the responsiveness of this PROM to therapeutic approaches.^35^ Thus, the use of PROMs such as HALT allows dentists to better understand how oral conditions influence patients’ physical and mental health.^8,17^

According to Miotto et al.,^29^ among oral health-related variables, halitosis was considered the condition that most negatively impacted QoL. Bad breath causes anxiety, depression, and significant impairment among daily activities, well-being, and social interactions.^3,36^ Halitosis can result in negative emotional and psychological consequences, such as decreased self-esteem, increased insecurity, and social withdrawal.^37^ Our data corroborate these findings as they highlight the impact of halitosis on psychological and social aspects.

Regarding the individualized HALT analysis, a statistically significant difference was observed among the three division categories. Concerning physiological alterations, He et al.^38^ (2012) described the relationship between halitosis and "mouth breathing" or "throat infection/tonsillitis" as weak. These studies proposed that these factors are causes rather than consequences of halitosis. By contrast, "social impact" and "emotional and self-esteem limitations" are more commonly associated with worse QoL in patients with halitosis.^1^

Although the target sample size was achieved, it remains small, underscoring the study's preliminary nature, in which the OH intervention significantly reduced self-reported halitosis, plaque, and BOP. Block randomization ensured a balanced distribution given the high prevalence and impact of smoking on periodontal status.^39^ Significant variables are expected to remain relevant in larger studies, with narrower confidence intervals, whereas nonsignificant results here should not be interpreted as the absence of effect. Future studies with larger and powered samples are needed to confirm these findings.

Interventions that enhance patient motivation and adherence to OH practices are essential for maintaining oral health.^8,21^ This study underscores the value of personalized OH guidance before and during professional therapy to improve participation and achieve effective, lasting outcomes in halitosis and periodontitis management.^4^ Low-cost personalized OH instruction before complex dental procedures can be readily integrated into resource-limited healthcare systems from a public health perspective, offering substantial benefits for OHRQoL.^27^

Finally, the limitations of this study include the small sample size. In addition, the halitosis findings were derived from secondary analyses primarily powered for probing depth, making the results exploratory. Future RCTs with halitosis as the primary outcome are warranted to robustly determine the actual effect of the OH intervention on self-reported halitosis. Although some nonsignificant results may reflect limited power, the intervention's effectiveness is supported, and a larger sample would improve precision. The Hawthorne effect in the NIG may have reduced power, but increasing participant numbers is expected to preserve significant effects.

The strengths of this study include the controlled trial design with blinded evaluation. This approach reduces bias risks, the use of a validated halitosis-specific PROM, and an individualized OH intervention protocol.^40^ These new findings reinforce the relevance of the relationship between improved personalized OH and self-reported halitosis.

## Conclusions

This preliminary study demonstrates that a strict OH phase can reduce self-reported halitosis and improve QoL in patients with periodontitis. Further studies with larger, more diverse populations are needed to confirm these findings and clarify the effectiveness of strict OH protocols as a strategy to mitigate the psychosocial impact of halitosis.

## Data Availability

The datasets generated during and/or analyzed during the current study are available from the corresponding author on reasonable request.
